# Mesenchymal Epithelial Transition Factor Signaling in Pediatric Nervous System Tumors: Implications for Malignancy and Cancer Stem Cell Enrichment

**DOI:** 10.3389/fcell.2021.654103

**Published:** 2021-05-13

**Authors:** Amanda Rose Khater, Tamara Abou-Antoun

**Affiliations:** Department of Pharmaceutical Sciences, School of Pharmacy, Lebanese American University, Byblos, Lebanon

**Keywords:** pediatric nervous system tumors, cancer stem cells, mesenchymal epithelial transition factor signaling, hepatocyte growth factor/scatter factor, therapeutic resistance

## Abstract

Malignant nervous system cancers in children are the most devastating and worrisome diseases, specifically due to their aggressive nature and, in some cases, inoperable location in critical regions of the brain and spinal cord, and the impermeable blood-brain barrier that hinders delivery of pharmaco-therapeutic compounds into the tumor site. Moreover, the delicate developmental processes of the nervous system throughout the childhood years adds another limitation to the therapeutic modalities and doses used to treat these malignant cancers. Therefore, pediatric oncologists are charged with the daunting responsibility of attempting to deliver effective cures to these children, yet with limited doses of the currently available therapeutic options in order to mitigate the imminent neurotoxicity of radio- and chemotherapy on the developing nervous system. Various studies reported that c-Met/HGF signaling is affiliated with increased malignancy and stem cell enrichment in various cancers such as high-grade gliomas, high-risk medulloblastomas, and MYCN-amplified, high-risk neuroblastomas. Therapeutic interventions that are utilized to target c-Met signaling in these malignant nervous system cancers have shown benefits in basic translational studies and preclinical trials, but failed to yield significant clinical benefits in patients. While numerous pre-clinical data reported promising results with the use of combinatorial therapy that targets c-Met with other tumorigenic pathways, therapeutic resistance remains a problem, and long-term cures are rare. The possible mechanisms, including the overexpression and activation of compensatory tumorigenic mechanisms within the tumors or ineffective drug delivery methods that may contribute to therapeutic resistance observed in clinical trials are elaborated in this review.

## Introduction

Nervous system tumors comprise the second most frequent malignancy (after leukemia) diagnosed in children and also account for the highest cancer-related mortality (Ostrom et al., 2019). Gliomas account for nearly half of all childhood central nervous system (CNS) tumors, followed by medulloblastomas (MBs) and other embryonal and neuroectodermal tumors such as the extracranial neuroblastomas (NBs; Bahmad et al., 2019, 2020). These malignant cancers express immense cellular heterogeneity and specific tumor microenvironmental niches such as brain-resident cell types, the selective permeability of the blood-brain barrier (BBB), and various immune-suppressive factors that are some of the myriad of factors that impede successful treatment attempts (Quail and Joyce, 2017). Moreover, diffuse intrinsic pontine gliomas (DIPGs), a sub-group of high-grade gliomas (HGG), commonly arise in the pons and midline region of the brainstem, a highly critical area in the brain where vital centers for respiration and cardiovascular function reside. This location makes these cancers inoperable due to the high risk of possible fatalities that may arise during surgical resection. In addition, the highly selective and impermeable BBB, composed of tight junctions and a chemical milieu that protects the neuronal tissue from damaging insults, which may arrive *via* the blood stream, further inhibits the delivery of the cancer therapeutics to the tumor site (Gerstner and Fine, 2007).

The standard of care therapy for the high-risk brain tumors includes surgical resection followed by radio- and/or chemotherapy, whereas the low-risk neuroblastomas and low-grade gliomas require only surgical resection. Moreover, the more aggressive gliomas, such as the diffuse midline gliomas, and the CNS germline tumors generally cannot undergo surgical resection (Vitanza and Monje, 2019) due to their infiltrative nature. Unfortunately, despite major advances and innovative approaches in the latest therapeutic interventions, the heterogenic nature of these tumors, the tumorigenic micro-environmental niche influences, the inoperable nature of some tumors such as diffuse midline gliomas, and the impermeable BBB, make malignant relapse a main hurdle to complete cures. In addition, children who survive these therapeutic interventions will consequently suffer from the toxic effects induced by the chemo- and/or radiotherapy on their developing nervous system (Brinkman et al., 2016). More targeted and less catastrophic therapies with better BBB penetration are highly desired in order to provide better cures for children with these devastating cancers.

The hepatocyte growth factor/scatter factor (HGF/SF) and its receptor tyrosine kinase (RTK) protein c-Met activate a signal transduction cascade that ultimately induces cellular proliferation, migration, invasion, and evasion of apoptosis. This signaling cascade is essential during development and tissue regeneration, such as when hepatocytes undergo regeneration (Yamada et al., 1994); however, cells undergoing oncogenic transformation may manipulate this signaling cascade to their advantage to advance their malignancy (Zhang and Vande Woude, 2003). In support of this, various studies have implicated the involvement of HGF/c-Met in many human malignancies including high-grade brain tumors (Abounader and Laterra, 2005) such as glioblastomas (GBM; Koochekpour et al., 1997), DIPGs (Paugh et al., 2011; Puget et al., 2012), high-risk NBs (Hecht et al., 2004), and MBs (Binning et al., 2008).

## Biological Function of the Hepatocyte Growth Factor/Mesenchymal Epithelial Transition Factor Signaling Cascade

The RTK, MET (c-Met), and its ligand, HGF/SF (Bottaro et al., 1991), play crucial roles in cell survival, growth, and migration (Owen et al., 2010). The Met receptor is a transmembrane heterodimeric RTK commonly expressed on epithelial cells of all tissues. Its induction can be triggered by various factors including HGF, epidermal growth factor (EGF), interleukins 1 and 6 (IL-1/6), as well as tumor-necrosis factor-alpha (TNF-α; Boccaccio et al., 1994). Upon ligand binding, c-Met undergoes dimerization and phosphorylation of Tyr1230, Tyr1234, and Tyr1235, which subsequently leads to auto-phosphorylation of the carboxy-terminal bidentate substrate-binding sites Tyr 1,349 and Tyr1356 (Weidner et al., 1996). This forms docking sites for various other molecules to become recruited and phosphorylated such as the growth factor receptor-bound protein 2 (GRB2), Grb2-associated adaptor protein 1 (GAB1), phospholipase C (PLC), proto-oncogene SRC, SRC homology protein tyrosine phosphatase 2 (SHP2), phosphatidylinositol 3-kinase (PI3K), and CRKL [v-crk sarcoma virus CT10 oncogene homolog (avian)-like] (Paliouras et al., 2009). Signaling pathways driven by c-Met activation are mediated *via* the PI3K-AKT and the rat sarcoma oncogene homolog (RAS)-mitogen activated protein kinase (MAPK) pathways that induce activation of cell division control protein 42 (CDC42), which in turn modulates cell survival. CDC42 also regulates cell migration and adhesion *via* interaction with RAP1 and RAC1 that act on integrins and cadherins (Birchmeier et al., 2003).

c-Met signaling is reported to be essential in epithelial-to-mesenchymal transition (EMT) required during the developmental process where extensive cellular migration occurs (Bladt et al., 1995). In cancer cells, activation of c-Met results in hijacking of the EMT process to enhance invasive metastasis and the functional characteristics of a stem-like subset of cells within the bulk tumor (Marona et al., 2019).

## Cross-Talk Between Mesenchymal Epithelial Transition Factor and Other Tumorigenic Pathways

**Figure 1:**
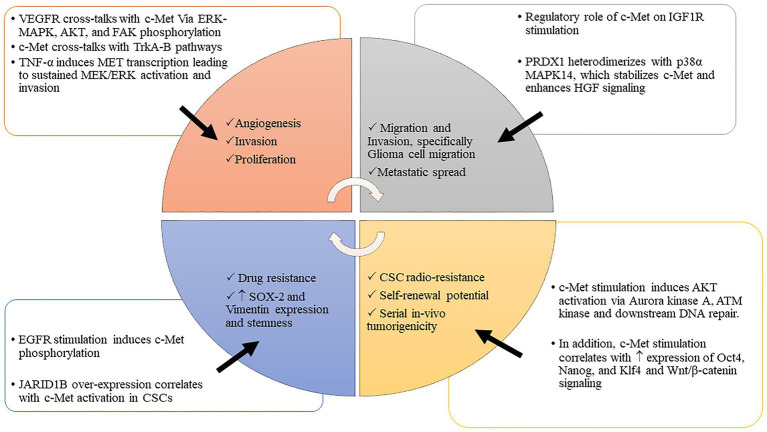
c-MET interacts with various tumorigenic processes to drive malignancy. The interplay between c-MET and various tumorigenic pathways promotes cancer invasion, migration, proliferation, stem cell enrichment, and therapeutic evasion. Abbreviations: c-MET, mesenchymal epithelial transition factor; WNT/β-catenin, wwingless-related integration site/β-catenin pathway; Oct 4, octamer-binding transcription factor 4; Nanog, transcription factor that is involved in the self-renewal of embryonic stem cells; Klf 4, Krüppel-like factor; JARID 1B, Jumonji C-domain-containing histone demethylase 1B; HGF/SF, hepatocyte growth factor/scatter factor; VEGFR, vascular endothelial growth factor receptor; IGF 1R, insulin-like growth factor 1 receptor; EGFR, epidermal growth factor receptor; PI3K-AKT, phosphoinositide 3-kinase-AK strain transforming; MAPK, mitogen-activated protein kinase; TrKA-B, tropomyosin receptor kinase A-B; PDRX1, peroxiredoxin 1; TNF-α, tumor necrosis factor α; MEK/ERK, MAPK/ERK kinase/extracellular receptor kinase; ATM kinase, ataxia-telangiectasia-mutated (ATM) protein kinase; CSCs, cancer stem cells; p-38α, p38 MAPK family (MAPK14); SOX2, sex-determining region Y-box 2.

The delicate balance of critical biologic processes, such as cell proliferation, migration, and differentiation is largely controlled by RTKs. Abnormalities in protein expression of these RTKs in tumor cells dysregulate multiple downstream signaling cascades that drive cell proliferation, invasion, metastasis, and angiogenesis (Figure 1). This dysregulation of RTK networks has been shown to correlate with poor patient survival in GBM and other malignancies (Yang et al., 2015).

Cross-talk between c-Met signaling with other tumorigenic pathways has been reported, including direct interactions with the insulin-like growth factor receptor-1 (IGF1R) in pancreatic cancer (Bauer et al., 2006), transforming growth factor-beta (TGF-β) in fibroblasts (Bhowmick et al., 2004), and in DIPG (Saratsis et al., 2014) and GBM (Papa et al., 2017), wingless-related integration site (WNT) in liver and bladder cancer (Monga et al., 2002; Khoury et al., 2005) and GBM (Kim et al., 2013), TNF-α in colorectal cancer (Bigatto et al., 2015), the vascular-endothelial growth factor receptor (VEGFR; Zhang et al., 2003), the fibroblast growth factor receptor (FGFR; Harbinski et al., 2012), the nonseleno peroxidase peroxiredoxin 1 (PRDX1; Wirthschaft et al., 2018), and epidermal growth factor receptor (EGFR) pathways. These interactions promote cancer progression and malignancy in various ways. For example, EGFR stimulation leads to c-Met phosphorylation and subsequent drug resistance in lung cancer that is reversed with dual c-Met and EGFR inhibitors (Engelman et al., 2007). EGFR and c-MET are co-expressed in multiple cancers including GBM, supporting the notion that their downstream signaling contributes toward this malignant phenotype.

Velpula et al. (2012) demonstrated that GBM cell lines (U251 and 5,310) cultured with or without human umbilical cord blood stem cells (hUCBSCs) exhibited differential response to treatment with EGFR inhibitors, erlotinib, and gefitinib, used in combination with the c-Met inhibitor PHA-665752. Specifically, cells that were co-cultured with hUCBCSc exhibited enhanced anti-tumor response to the abovementioned inhibitors and exhibited a reduction in EGFR phosphorylation and a 50% reduced expression of EGFR, c-MET, β-catenin, and STAT-3. Functionally, co-culturing with hUCBSCs inhibited cellular invasion and wound healing in the GBM cell lines and patient-derived GBM cells implicating the added efficacy of these stem cells to receptor tyrosine kinase-targeted therapy. Moreover, EGFR and c-Met co-localized in glioma cells and GBM, indicating a possible relationship between EGFR and c-Met signaling pathways (Velpula et al., 2012). Furthermore, Bender et al. (2016), identified fusion genes involving the MET oncogene in 10% of pediatric GBM that stimulated the MAPK pathway and abrogated the cell cycle regulation in these cancers leading to aggressive *in vivo* clonogenic growth. The combined targeting of c-Met and EGFR pathways could be a promising tool for GBM and a possible treatment option to explore for the aggressive and malignant nervous system tumors.

Overexpression or mutation in the WNT/β-catenin pathway is known to play a significant role in cancer transformation; c-Met is a direct transcriptional target of WNT/β-catenin in colon cancer cell lines (Boon et al., 2002). Moreover, in liver and bladder cancer, HGF/SF induces nuclear translocation of β-catenin-T-cell factor (TCF) with subsequent transcription of their target genes (Monga et al., 2002), further implicating c-Met and WNT pathways in tumorigenic processes. In addition, c-Met was reported by Kim and colleagues to signal through the WNT/β-catenin pathway in GBM stem cells (Kim et al., 2013).

Signaling through the HGF/c-Met pathway was reported to trigger the upregulation and secretion of the pro-angiogenic VEGF in malignant glioma cells thereby inducing the proliferation of neuromicrovascular endothelial cells within the brain cancers *via* a paracrine and autocrine loop signaling (Moriyama et al., 1998). The VEGFR-c-Met cross-talk utilizes the same pathways, ERK-MAPK, AKT, and FAK, through the common signaling intermediate SHC driving angiogenesis (Zhang et al., 2003). In addition, HGF/SF inhibits thrombospondin, a negative regulator of angiogenesis, thereby inducing VEGFA and angiogenesis. In support of this, Nakagawa et al. (2010) reported that dual inhibition of c-Met and VEGFR2 led to tumor regression, reduced angiogenesis, and prolonged survival of tumor-bearing mice in an *in vivo* model of gastric cancer (Nakagawa et al., 2010). Various other cancer xenograft models have shown that HGF/SF-MET signaling enhances cancer invasion and metastasis, while inhibition of such signaling leads to reduced invasive potential and metastasis (Bardelli et al., 1998; Jeffers et al., 1998; Gallego et al., 2003). To better understand the mechanism of these interactions, more recent studies have further demonstrated the cross-talk between MET and VEGFR2 in GBM that are resistant to anti-angiogenic therapy. In response to the anti-angiogenic therapy in GBM, VEGFR2 was found to phosphorylate MET leading to invasion and therapeutic resistance *via* activation of the signal transducer and activator of transcription 3 (STAT3; Lu et al., 2012; Jahangiri et al., 2013).

In another recent study, Bigatto et al. (2015) reported TNF-α to be highly correlated with increased MET and HGF expression in human colorectal cancers. The ability of TNF-α to drive invasion was accomplished by inducing transcription of MET *via* an NF-κB signaling. Subsequently, this led to sustained MEK/ERK activation, Snail accumulation, and E-cadherin downregulation. The pro-invasive capacity of TNF-α was markedly reduced with small molecule inhibition or transcriptional silencing of MET, implicating an important cross-talk between these tumorigenic players (Bigatto et al., 2015).

In a more recent report, Wirthschaft et al. (2018) showed that PRDX1 interacts with c-Met in a pre-clinical IDH-WT/mutant glioma murine model (Wirthschaft et al., 2018). PRDX1 stimulated infiltrative growth of IDH-wild-type gliomas. Expression of PDRX1 had a stabilizing effect on c-Met. Specifically, PRDX1 forms a heterodimer with p38α MAPK14, stabilizing phospho-p38α in glioma cells and enhancing HGF-mediated signaling. This, in turn, stimulated actin cytoskeleton dynamics, enhancing glioma cell migration. Moreover, PRDX1 enhanced *in vivo* glioma brain invasion, but reducing its expression increased survival in mouse glioma models.

## Dysregulation of Mesenchymal Epithelial Transition Factor/Hepatocyte Growth Factor in Pediatric Nervous System Cancers

### Medulloblastomas

Medulloblastomas comprise the most common malignant childhood brain tumors.

The standard of care therapy for MBs includes surgical resection and radiotherapy in children older than 3 years of age. For children under the age of three, where radiotherapy is not an ideal option due to the neurological impairment associated with this treatment, multi-target chemotherapy (including methotrexate, vincristine, cisplatin, and cyclophosphamide) is used in order to delay the need for radiotherapy until absolutely necessary (Robinson et al., 2018; Juraschka and Taylor, 2019). Despite much advancement in targeted therapeutics for this cancer, long-term consequences of radio and/or chemotherapy, including neurological impairment such as deficits in neurological processing speed, attention, and working memory (de Spéville et al., 2021), necessitate the need for better targeted therapy with reduced off-target effects.

Four subgroups have been identified in MB based on gene expression profiling showing differences in karyotype, histology, and prognosis, which include the Sonic Hedgehog (SHH)-subgroup (named after the pathway believed to be the prime driver of tumor initiation in this subgroup) that arises from granule precursors of the external granule layer, WNT-subgroup that arises from the progenitor cells in the lower rhombic lip, subgroup-3 that arises from the neural stem cells, and subgroup-4 that arises from the unipolar brush cells (Taylor et al., 2012; Juraschka and Taylor, 2019). The WNT-subgroup rarely metastasizes and has the best prognosis, subgroup-3 readily metastasizes and is affiliated with the poorest prognosis, the SHH-subgroup presents with intermediate prognosis and is commonly non-metastatic, whereas subgroup-4 usually presents with frequent metastasis and possesses an intermediate prognosis. The SHH-subgroup tumors exhibit high levels of *MYCN* expression, the WNT-subgroup and subgroup-3 tumors exhibit high levels of *MYC* expression, whereas subgroup-4 tumors exhibit relatively low expression of both *MYC* and *MYCN* (Northcott et al., 2011).

Interaction between the c-Met and SHH pathways is reported in MB. Earlier studies showed a co-stimulatory role played by the SHH pathway and HGF in MB initiation and growth. Of the four MB subgroups, HGF-induced c-Met activation was most pronounced in the SHH subgroup. MB cell lines from the SHH subgroup activated c-Met *via* MAP4K4 leading to increased cell migration. This increase in metastatic potential was dependent on the c-Jun N-terminal kinase (JNK) pathway (Santhana Kumar et al., 2015). More recently, Tripolitsioti et al. (2018) revealed the mechanism by which c-Met induces metastatic invasion in MBs. The authors report that MAP4K4 acts as a downstream mediator of c-Met-triggered invasion by regulating F-actin dynamics, integrin β1 activation, and c-Met endocytosis (Tripolitsioti et al., 2018).

### Pediatric High-Grade Gliomas

Pediatric HGGs, which include GBM, anaplastic astrocytoma, and DIPG, are highly infiltrative and diffuse in nature making them largely inoperable with radiotherapy being the mainstay of treatment (Ostrom et al., 2019). Those that can be surgically removed are often further treated with conventional chemotherapy and radiotherapy. They have been reported to harbor various molecular aberrations that differ from those seen in adult gliomas (Abou-Antoun et al., 2017). The majority of pediatric brainstem tumors are DIPG, representing 75–80% of pediatric brainstem tumors. The majority of children are diagnosed with DIPG between the ages of 5–10 years with a median age at diagnosis of 6–7 years (Warren, 2012). The median survival is 11 months, whereas overall 2- and 3-year survival is reported by Hoffman et al. (2018) to be 9.6 and 4.3%, respectively, whereas the 5-year survival is only 2.2%.

The cell of origin of high-grade gliomas and particularly DIPG seems to be the neural progenitor cells that gain signature tumorigenic mutations, where the histone H3K27M mutation serves as the pillar, around which other mutations, including the TP53 and ACVR1, develop giving rise to transformed cancerous cells (Nikbakht et al., 2016).

Pathways that govern cell cycle regulation were predominantly altered in pediatric HGG with mutations in TP53 and PPM1D, repression of CDKN2A (Mistry et al., 2015), or homozygous deletion of *ADAM3A* (Barrow et al., 2010). In addition, activating mutations in EGFR (Brennan et al., 2013) or its downstream protein neuroblastoma RAS viral oncogene homolog (NRAS; Gits et al., 2018), Ki-ras2 Kirsten rat sarcoma viral oncogene homolog (KRAS), v-raf murine sarcoma viral oncogene homolog B1 (BRAF; Schiffman et al., 2010), and PI3KCA have been reported (Diaz and Baker, 2014). In addition to mutations, amplifications of EGFR, the platelet-derived growth factor receptor (PDGFR)/KIT proto-oncogene, receptor tyrosine kinase (KIT), and MET were shown to be present, such as amplification of the PDGFRA in HGG and DIPG (Paugh et al., 2011), which correlated with the activation of the RTK PI3K/AKT/mTOR signaling pathway. While this may not necessarily imply a causative effect, it suggests that EGFR/PDGFR/MET amplification and activation of PI3K/AKT/mTOR may play a significant role in DIPG (Paugh et al., 2011). Moreover, c-MET was reported to be the second most frequently amplified gene in DIPG following PDGFRA (Paugh et al., 2011; Puget et al., 2012) and affiliated with poor survival (Dufour et al., 2020). The International Cancer Genome Consortium: Ped. Brain Tumor Project reported a recurrent drug-targetable *MET* fusion gene in pediatric GBMs. The study employed the use of genetic analysis in 53 pediatric GBMs as well as in five *in vitro* tumor model systems and reported a previously unidentified *MET* oncogene-affiliated gene fusion in approximately 10% of cases. The presence of these *MET*-fusions triggered MAPK stimulation and, when present in tumors with deregulated cell cycles, gave rise to aggressive glial tumors in an *in vivo* model. The malignant nature of this model was alleviated with reduced tumor burden using Met-specific inhibitors in an *in vivo* xenograft model (Bender et al., 2016).

Mackay et al. (2017) described a subgroup of HGG that was wild type (WT) for H3/IDH1 that can further be subdivided into two poor-outcome groups: one designated as WT-B and driven by *EGFR*/*MYCN* and cyclin-dependent kinase 6 (*CDK6*), and the other designated as WT-C and driven by *PDGFRA*/*MET*. *The WT-C subgroup, which harbored the PDGFRA* and *MET* amplifications also expressed enrichment for chromosome 1p and 20q loss and 17q gain. The gene signature of this group highly resembled that of the adult GBM-defined “Proneural” group, and the median survival of patients within this group was approximately 18 months (Mackay et al., 2017), while the methylation-based classifications of these two groups resembled others such as the PDGFRA versus EGFR versus MYCN (Korshunov et al., 2017) and the “GBM_pedRTK” versus “GBM_MYCN” versus “HGG_MID” (molecularneuropathology.org/mnp). They are uniquely defined here due to their diverse anatomical locations and integration with sequencing data.

Ohba et al. (2019) reported data on c-MET expression in adult GBMs according to the new 2016 WHO classification of glioma diagnosis. Not only did they reassess the percentage of c-MET-positive cells present in different GBM types, but they also found that oligodendrogliomas, a previously c-MET-negative cancer, also expressed a substantial level of the marker. While no correlation was found between c-MET and the MIB-1 index, an association between c-MET expression and the overall survival rate in different gliomas was identified, although further studies will need to be conducted to further elaborate on the matter. Another prognostic factor that was determined to be the O[6]-methylguanine-DNA methyltransferase (MGMT) promoter region is independent from c-MET expression. The attempt to identify c-MET as a surrogate marker for different GBM subtypes was found to be ineffective in distinguishing astrocytic tumors with IDH mutation from oligodendroglial tumors and futile to diagnose GBM owing to its inconsistent expression on a case-by-case basis (Ohba et al., 2019).

### Neuroblastomas

Neuroblastoma is the most common extracranial solid tumor in children, which arises from the neural crest cells, destined to differentiate into the sympathetic chain ganglia and adrenal medulla, which are components of the sympathetic nervous system (Abou-Antoun et al., 2018). NB presents with a wide clinical heterogeneity, where in some cases, it undergoes spontaneous regression, whereas in other cases, it progresses aggressively leading to mortality (Chakrabarti et al., 2012). While patients with low- and intermediate-risk NB respond very well to surgery alone (De Bernardi et al., 2008), the standard of care for patients with high-risk NB includes multi-agent chemotherapy induction followed by surgical resection, consolidative high-dose chemotherapy with autologous stem cell transplant, post-transplant radiotherapy, and post-consolidation treatment with biological agents and immunotherapy (Pinto et al., 2015). Amplification of the bHLH transcription factor MYCN, a member of the MYC family of phosphoproteins, confers poor prognosis in patients with an overall survival rate after diagnosis of <50%. This amplification and subsequent aberrant expression of MYCN in NB patients put them in the high-risk NB group because their tumors progress aggressively, exhibit a non-differentiated, stem-like cellular phenotype, evade apoptosis, and are chemo- and radiotherapy resistant (Zaatiti et al., 2018).

**Figure 2:**
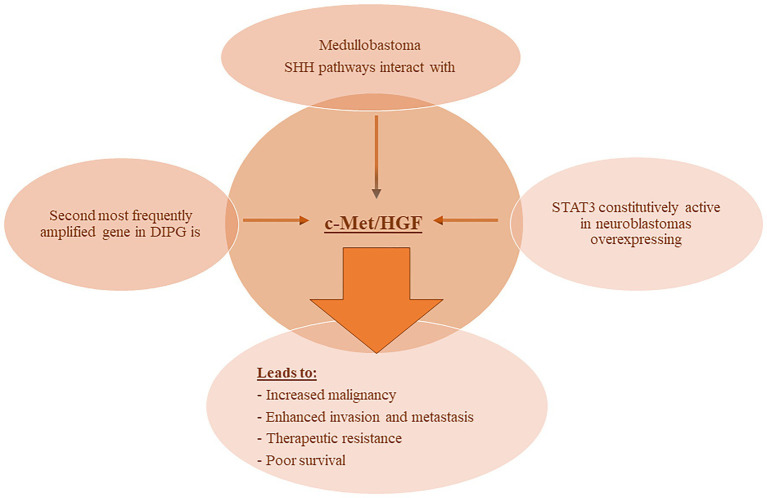
c-MET involvement with pediatric NS tumors. Various reports have demonstrated the involvement of c-MET/HGF signaling in pediatric nervous system tumors leading to enhanced malignancy and metastatic potential, therapeutic evasion, and poor survival. Abbreviations: c-MET, mesenchymal epithelial transition factor; HGF, hepatocyte growth factor; DIPG, diffuse intrinsic pontine glioma; SHH, Sonic Hedgehog; STAT3, signal transducer and activator of transcription 3.

The involvement of c-Met and its ligand HGF in NB have been reported in various studies. In one report, Hecht et al. (2004), demonstrated that HGF binding to c-Met induced auto-phosphorylation of the receptor and subsequent activation of the downstream MAPK signaling cascade. This signaling induced the expression and proteolytic activity of matrix metalloproteinase-2 and tissue-type plasminogen activator in NB cells thereby enhancing their ability to degrade the extracellular matrix and invade nearby tissues. Moreover, STAT3 was constitutively active in NB cells overexpressing c-Met, and its inhibition or the inhibition of MAPK reduced the invasive and malignant capacity of the c-Met expressing NB cells (Hecht et al., 2004). More recently, one report demonstrated the added benefit of combining both VEGFR and c-Met inhibitors in an NB *in vivo* orthotopic model on reduction of tumor growth, inhibition of neovascularization and metastasis, and enhanced apoptosis (Daudigeos-Dubus et al., 2017). Refer to Figure 2 for an illustration of c-Met/HGF’s involvement in the malignancy and therapeutic evasion of various pediatric nervous system tumors.

## Met/Hepatocyte Growth Factor and Epigenetic Regulation

Epigenetic dysregulation has been affiliated with many brain cancers, including adult GBM (Ohba et al., 2019), pediatric HGG (Paugh et al., 2011), MBs (Santhana Kumar et al., 2015), and the extracranial NBs (Pinto et al., 2015), and many therapeutic approaches are currently aimed at targeting these epigenetic machineries. Whether c-Met plays a prominent role in this dysregulation leading to therapeutic failure in clinical trials is yet to be determined. Studies in lung cancers have shown an overexpression of the H3K4 histone demethylase protein, Jumonji AT-rich interactive domain 1 (JARID1) in the cancer stem cell (CSC) population (Kuo et al., 2018). Furthermore, this overexpression of JARID1B correlated with c-Met activation in CSCs. When JARID1B was silenced, c-Met expression was simultaneously reduced as was the expression of the stem markers SOX2 and vimentin. In addition, tumorsphere self-renewal, invasion, and proliferation were all inhibited after JARID1B knockdown implicating its importance in tumorigenic mechanisms involving the c-Met pathway. Somatic mutations in the epigenetic regulator genes including JARID1A/B have been reported in pediatric MB (Robinson et al., 2012) and NB (Kuo et al., 2015), whereas HGGs were reported to harbor mutations in the JARID1C (Fontebasso et al., 2013).

The involvement of c-Met and its ligand HGF/SF in epigenetic dysregulation in MBs has been reported. Kongkham et al. (2008) conducted an epigenome-wide screen in MB cell lines, using DNA methylation profiling to identify genes aberrantly silenced by promoter hypermethylation. The tumor suppressor serine protease inhibitor kunitz-type 2 (SPINT2/HAI-2), an inhibitor of HGF/c-Met signaling, was silenced by promoter methylation (Kongkham et al., 2008). SPINT2 gene expression was downregulated, as expected, given the promoter methylation. Correspondingly, c-Met expression was upregulated. When SPINT2 was transfected into MB cell lines to increase SPINT2 expression, the resulting cell lines showed a decrease in cell proliferation and migration. Mice xenografted with a SPINT2 overexpressing MB cell line had a mean overall survival of 64.0 days compared with mice xenografted with empty vector (29.3 days; Kongkham et al., 2008). This indicates that one possible mechanism of c-Met overexpression in NB is by epigenetic modification of promoter methylation.

**Figure 3:**
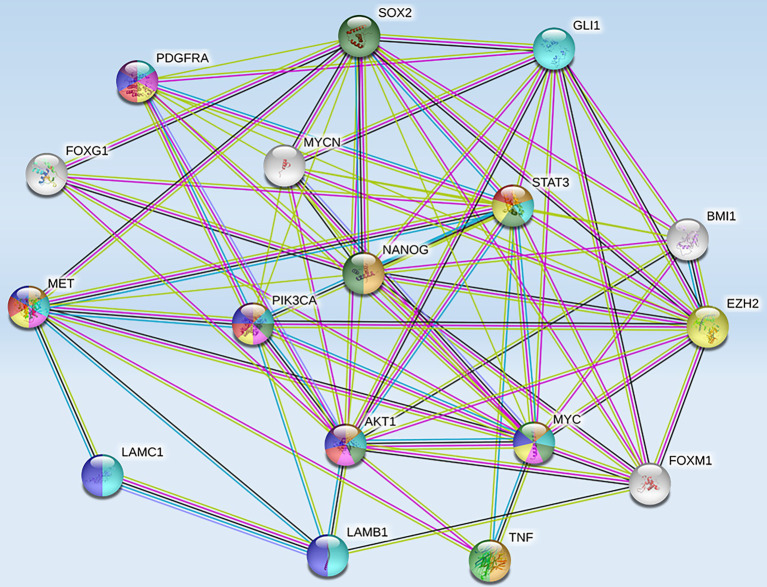
Protein-protein interactions between MET and tumorigenic markers in brain CSCs. String functional association networks reveal known interactions between MET and other tumorigenic players identified in brain CSCs. Known interactions were identified experimentally (pink lines), from curated databases (blue lines), and predicted interactions were identified as co-expressed proteins (black lines) and from text mining (green lines). Abbreviations: EZH2, enhancer of zeste homolog 2; SOX2, sex-determining region Y-box 2; PDGFRA, platelet-derived growth factor receptor A; GLI 1, glioma-associated oncogene homolog 1; FOXG 1, forkhead box protein G1; STAT 3, signal transducer and activator of transcription 3; BMI 1, B lymphoma Mo-MLV insertion region 1 homolog; PIK3CA, phosphatidylinositol-4,5-bisphosphate 3-kinase catalytic subunit alpha; NANOG, transcription factor that is involved in the self-renewal of embryonic stem cells; LAMC 1, laminin subunit gamma-1 precursor; AKT 1, AK strain transforming 1; MYC, proto-oncogene, bHLH transcription factor; MYCN, v-myc myelocytomatosis viral-related oncogene, neuroblastoma-derived; FOXM 1, forkhead box M1; LAMB 1, laminin subunit beta-1; TNF, tumor necrosis factor.

High-risk NB was also reported to harbor epigenetic dysregulation including elevation of enhancer of zeste homolog 2 (EZH2), which is associated with enrichment of H3K27me3 at the promoters of tumor suppressor genes such as *CASZ1*, *RUNX3*, *NGFR* (p75), and *NTRK1* (TrkA; Wang et al., 2012). In addition, BMI1 is a direct transcriptional target of MYCN, which is also upregulated in the high-risk NB patients (Cui et al., 2006). Moreover, the tumor suppressor CHD5, a member of the chromatin remodeling family proteins, is reported to undergo loss of heterozygosity (LOH) in NB. *CHD5* contains a bivalent chromatin mark in stem cells, and in NB cells with LOH, DNA methylation silences the remaining allele (Fujita et al., 2008). Genome-wide expression microarray data derived from primary NB tumors identified an inverse correlation between *EZH2* and *CHD5* expression, but a direct correlation between EZH2 and DNA methytransferase-1 (*DNMT1*) expression indicating that this may lead to permanent gene silencing of critical developmental loci. Whether or not silencing of a single gene or entire pathways is required for NB initiation and progression is still under investigation. However, several hypermethylated genes have been consistently identified in NB, including HIC1, putative tumor suppressor genes (*RASSF1A*, *PRKCDBP*), differentiation genes (*HOXA9*), and apoptosis genes (*CASP8*, *APAF1*, and *TMS1*; Carén et al., 2011). Furthermore, MYCN amplification has been associated with activating histone chromatin modifications as well as with DNA methylation *via* its interaction with the CpG methyl-binding protein MeCP2, thereby suggesting its potential role in recruiting essential components for the methylation of DNA (Murphy et al., 2009). String functional protein network analysis revealed an intricate interplay between some of these proteins and MET (Figure 3), which were grouped into pathways highly involved in cancer progression, angiogenesis, invasion/migration, evasion of apoptosis, stem cell self-renewal, and therapeutic resistance (Table 1).

**Table 1:** Kyoto Encyclopedia of Genes and Genomes (KEGG) pathway analysis links MET to highly tumorigenic pathways.

Description	Count in gene set	False discovery rate
Proteoglycans in cancer	7 of 195	1.83e−08
Pathways in cancer	9 of 515	1.83e−08
Signaling pathways regulating pluripotency of stem cells	6 of 138	7.64e−08
Central carbon metabolism in cancer	5 of 65	8.93e−08
MicroRNAs in cancer	6 of 149	8.93e−08
EGFR tyrosine kinase inhibitor resistance	5 of 78	1.67e−07
PI3K-Akt signaling pathway	7 of 348	2.00e−07
Jak-STAT signaling pathway	5 of 160	2.64e−06
MAPK signaling pathway	5 of 293	3.07e−05
FoxO signaling pathway	4 of 130	3.24e−05
Glioma	3 of 68	0.00013
TNF signaling pathway	3 of 108	0.00034
Transcriptional misregulation in cancer	3 of 169	0.00097
mTOR signaling pathway	3 of 148	0.00070
VEGF signaling pathway	2 of 59	0.0027
TGF-beta signaling pathway	2 of 83	0.0046

The Kyoto Encyclopedia of Genes and Genomes (KEGG) database, used to integrate and interpret large-scale data from genome sequencing and other high-throughput experimental techniques such as proteomics analysis, has revealed the top pathways in which these interactions are involved. Abbreviations: EGFR, epidermal growth factor receptor; PI3K-AKT, phosphoinositide 3-kinase-AK strain transforming; Jak-STAT, Janus kinase and signal transducer and activator of transcription; MAPK, mitogen-activated protein kinase; FOXO, class O of forkhead box transcription factors; TNF, tumor necrosis factor; mTOR, mechanistic (formerly “mammalian”) target of rapamycin; VEGFR, vascular endothelial growth factor receptor; TGF-β, transforming growth factor β.

## Dysregulated Met/Hepatocyte Growth Factor in Nervous System Cancer Stem Cells

CSCs comprise a small sub-population of self-renewing, multi-potent cells that reside within the bulk of the tumor. This sub-population is phenotypically and functionally similar to normal stem cells, but can drive tumor growth and recurrence (Lathia et al., 2015). CSCs are characterized by sustained self-renewal potential, limitless proliferation, multi-potency, and the ability to initiate tumors and evade conventional therapies (Lathia, 2013), while the non-stem cancer cells that comprise the bulk of the tumor often respond well to conventional therapy and fail to recapitulate the heterogenic nature of the original tumor. CSCs were characterized from various patient-derived tumors including breast, brain, colon, and ovarian (Abou-Antoun et al., 2017). Unlike the bulk, non-stem cancer cells, the CSC sub-population is able to recapitulate the original tumor in serial *in vivo* tumor growth models and resist conventional therapeutic approaches.

Stem cell markers in nervous system tumors have been utilized to enrich the CSC population for functional studies and they include: CD15, CD133, CD49f, CD36, CD44, L1CAM, A2B5, SOX2, musashi-1, BMI1, EGFR, and Nestin (Singh, 2013; Abou-Antoun et al., 2017; Prager et al., 2019; Bahmad et al., 2020). Moreover, the epigenetic aberrations identified in childhood brain CSCs included H3K27M, BMI1, FOXG1, SOC2, Musashi-1 ATRX, BMP1, and EZH2, whereas those identified in adult brain CSCs included SOX2, FOXM1, FOXG1, NANOG, STAT3, GLI1, ASCLI, ZFX, ZFHX4, HOXA10, EZH2, and BMI1. The molecular drivers common to both adult and pediatric brain CSCs were NOTCH, PI3K/AKT/MAPK, Sonic Hedgehog, and WNT/β-catenin, whereas MYCN was identified as a specific molecular driver in pediatric brain CSCs, and TGF-β, VEGFR, EGFR, FACT, and HIF2α were specific molecular drivers of adult brain CSCs (Abou-Antoun et al., 2017; Prager et al., 2019; Draaisma et al., 2020).

The interplay demonstrated between c-Met and these pathways (Figure 3) implicates an intricate network of communication between these tumorigenic players that warrants further investigation. As illustrated in Figure 3 and outlined in Table 1, the signaling interaction between MET and the molecular drivers/epigenetic aberrations identified in adult and pediatric brain CSCs involve highly tumorigenic, stem cell renewal (SOX2, Nanog, MYC), proliferation-enhancing/anti-apoptotic (PI3K/AKT/MAPK/mTOR), pro-angiogenic (VEGFR), and drug-resistance (EGFR inhibitor resistance) pathways. The Kyoto Encyclopedia of Genes and Genomes (KEGG) database, used to integrate and interpret large-scale data from genome sequencing and other high throughput experimental techniques, such as proteomics analysis, has revealed the top pathways in which these interactions are involved (Table 1). Moreover the count in gene set (meaning the number of proteins from our list that are found in the gene set affiliated with the designated pathway) and the false discovery rates (that control for a low proportion of false positives, thereby increasing statistical power and decreasing type I errors) point to the significant involvement of such proteins in these pathways.

While studies looking at MET expression in DIPG stem cells are scarce, a number of studies have investigated the involvement of c-Met signaling in adult GBM stem cells (GSCs). Tasaki et al. (2016) showed that GSCs have significantly upregulated c-Met expression and that targeting of c-Met led to reduced self-renewal and *in vivo* tumorigenicity (Tasaki et al., 2016). As mentioned, c-Met has been shown to promote survival, proliferation, and invasion of various cancers including GBM. De Bacco et al. (2012) demonstrated the affiliation of the MET oncogene in GBM stem-like neurospheres exhibiting typical mesenchymal and proneural signatures. These MET-positive GBM neurospheres also exhibited amplification and increased EGFR gene expression further implicating the cross-talk between c-Met and EGFR to sustain GSCs. Enhanced cellular proliferation, clonogenicity, migration/invasion, and increased expression of self-renewal markers were stimulated with HGF activation of the MET pathway (De Bacco et al., 2012). Moreover, c-Met was found to inhibit the effects of forced differentiation in GBM neurospheres *via* an interaction with Nanog, thereby enhancing GBM stem cell enrichment and self-renewal (Li et al., 2011). In support of the above findings, Joo et al. (2012) identified a sub-population (comprising 10–30%) of the bulk tumors with overexpression of MET in human primary GBM specimens that were preferentially localized in perivascular regions of human GBM biopsies (Joo et al., 2012).

**Figure 4:**
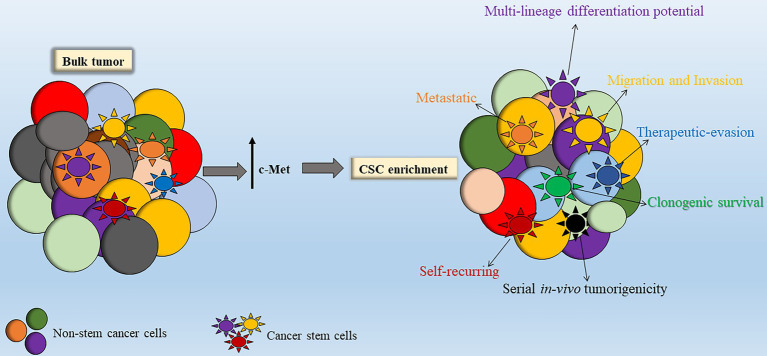
Stimulation of c-Met leads to enhanced cancer stem cell (CSC) properties. Within the bulk tumor resides a sub-population of stem-like tumor cells termed CSCs. Enhanced c-Met signal transduction leads to enrichment of the CSC sub-population within the bulk tumor. Abbreviations: c-Met: mesenchymal epithelial transition factor.

In addition, MET co-expression with CD133 and/or CD15 was two- to sevenfold that of non-MET expressing bulk tumor cells and MET-overexpression correlated with higher clonogenic and tumorigenic behavior and increased radio-resistance, indicating a possible affiliation of MET with CSC maintenance (Joo et al., 2012). c-Met signaling inhibition disrupted tumor growth and invasion *in vitro* and *in vivo*, indicating that c-Met activation may be required for GSC function (Joo et al., 2012), thereby suggesting a promising therapeutic target if also found to be true in HGGs such as DIPG among others (Figure 4).

GSCs from a genetically engineered mouse model of EGFR-driven GBM responded to anti-EGFR therapy by inducing high levels of c-Met expression. c-Met-expressing cells were preferentially localized in perivascular regions of mouse tumors consistent with their function as GSCs. They possessed self-renewing, clonogenic, and multi-lineage differentiation potential. GSCs were highly tumorigenic *in vivo* and resistant to radiotherapy. Furthermore, stimulation of c-Met signaling pathway increased expression of stemness transcriptional regulators Oct4, Nanog, and Klf4. The activation of these stemness markers was abrogated after pharmacological inhibition of MET signaling (Jun et al., 2014), indicating a possible interplay between MET and drivers of stem cell self-renewal.

The WNT/β-catenin signaling is active in GSC and is a downstream effector of c-Met. GSC expressed significantly higher levels of c-Met compared with bulk tumor cells. When c-Met signaling was increased or inhibited, there was a corresponding change in the expression of WNT/β-catenin signaling, which seemed to be related to β-catenin nuclear localization. Showing that WNT/β-catenin signaling is downstream of c-Met, inhibition of c-Met and the resulting loss of clonogenicity could be overcome with ectopic expression of β-catenin. As such, these data suggest that WNT/β-catenin signaling is a key downstream effector of c-Met signaling and contributes to the maintenance of GSCs in GBM (Kim et al., 2013). These observations warrant further exploration of c-MET-driven malignancy and stem cell enrichment in DIPG and high-risk pediatric nervous system tumors.

NOTCH and N-MYC have been demonstrated to be important pathways in DIPG. Monotherapy with the NOTCH inhibitor, γ-secretase, decreased cellular proliferation and increased apoptosis. This is further supported by experiments that depleted glioma CSCs *via* NOTCH inhibition using γ-secretase (Fan et al., 2006). In the case of N-MYC, the BET bromodomain inhibitor, JQ1 suppressed N-MYC expression. There was mild decrease in DIPG cell viability using JQ1, but when used in combination with the NOTCH inhibitor, the effect was significantly higher (Taylor et al., 2015). NOTCH signaling is a known stem cell driver and tumorigenic factor in CSCs. Further exploration of these heterogeneous subgroups in order to refine integrated molecular diagnostics to prioritize patient subpopulations for stratified treatment remains a priority.

Du et al. (2014) reported that knockdown of c-MET sensitized TRAIL-resistant NB and glioma cell lines to mesenchymal stem cell-delivered secretable form of TRAIL in both an *in vitro* and *in vivo* intracranial tumor model. In the anaplastic lymphoma kinase (ALK)/MYCN-driven NB, activation through the downstream signaling pathways of ALK (PI3K/AKT/MAPK, RAS/RAF/MEK, and JAK/STAT3) may enhance the propagation and maintenance of the stem cell-like sub-population in NB (Du et al., 2014). In support of this, lorlatinib (ALK/ROS1 inhibitor PF-06463922) demonstrated high efficacy in ALK-driven pre-clinical NB models that presented with primary crizotinib resistance. On the other hand, the use of single kinase inhibitors exhibited only transient benefit (Infarinato et al., 2016), implying the importance of using multi-targeted approaches to better treat the high-risk NB that harbor a sub-population of therapeutically resistant stem cells.

## Multi-Modal-Targeted Therapeutic Intervention Against Met Signaling

The commonly used approaches, to date, that inhibit c-Met signaling involve preventing HGF binding to the extracellular domain of c-Met with neutralizing antibodies or biological antagonists, preventing RTK phosphorylation using small-molecule inhibitors, and blocking the kinase-dependent signaling of c-Met through relevant signal transducers or downstream signaling components (Zhang et al., 2018). Most compounds are not solely targeting MET, but rather target one or more additional pathways in an attempt to prevent compensatory mechanisms that lead to therapeutic resistance. Therefore, we cannot be certain that the therapeutic benefit attained with these compounds is solely due to the specific targeting of MET, or due to the combinatorial targeting of the various pathways per compound.

Crizotinib is a small molecule inhibitor that targets both c-Met and ALK, whereas cabozantinib is a multi-tyrosine kinase inhibitor of c-Met, VEGFR2, AXL, KIT, TIE2, FLT3, and RET. Cabozantinib, in combination with the VEGFR1-3 inhibitor axitinib, inhibited NB cellular proliferation and migration *in vitro* and rescued the *in vivo* resistance to axitinib in an orthotopic mouse model. Mice treated with axitinib as sole therapy suffered from metastatic disease that was reduced with the combination of cabozantinib (Daudigeos-Dubus et al., 2017). This study supports the notion that perhaps the anti-tumor efficacy of the combination therapy was not specifically due to c-Met inhibition considering the multi-tyrosine kinase inhibition capacity of cabozantinib. One report demonstrated substantial tumor shrinkage and symptom relief in one pediatric patient bearing a MET-fusion-expressing glioblastoma treated with crizotinib (Bender et al., 2016). Unfortunately, relapsed lesions evaded this therapy highlighting the importance of multi-modality therapy to target compensatory upregulation of tumorigenic pathways is warranted in order to prevent malignant recurrence.

Tivantinib is a non-adenosine triphosphate (ATP) competitive c-Met inhibitor. To date, there are no reports of clinical trials using tivantinib in children with brain tumors. Only one report of a phase I trial in children with relapsed/refractory solid tumors reported no objective response with tivantinib treatment (Geller et al., 2017). Foretinib is another multityrosine kinase inhibitor of c-Met, c-ros oncogene (ROS), receptor d’origine nantain (RON), AXL, TIE2, and VEGFR2 (Faria et al., 2015). In a preclinical mouse xenograft model of SHH MB, foretinib showed promise by significantly reducing tumor growth, inhibiting metastatic spread, and increasing animal survival *in vivo*. A more recent study reported on the beneficial effects of foretinib treatment in entrectinib-resistant *NTRK1* fusion-positive tumors (including brain tumors) bearing the *NTRK1*-G667C mutation. Specifically, foretinib inhibited tyrosine receptor kinase A (TRK-A) phosphorylation in cells bearing the *NTRK1*-G667C mutation (Nishiyama et al., 2018), implicating the importance of combined therapy to overcome drug resistance. Again, here, it is crucial to emphasize that foretinib may be showing anti-tumor benefits *via* its multi-tyrosine kinase inhibition capacity and not specifically due to c-Met inhibition alone.

Humanized monoclonal antibodies that are directed against c-Met or neutralize HGF are onartuzumab and rilotumumab, respectively. In a multicenter phase II randomized, double-blind, placebo-controlled study of onartuzumab plus bevacizumab versus placebo plus bevacizumab in patients with recurrent GBM, combination therapy was beneficial for patients who presented with HGF overexpression or unmethylated O^6^-methylguanine-DNA methyltransferase (Cloughesy et al., 2017). More recently, Cruickshanks et al. (2018) treated MET inhibitor-resistant GBM cell lines with a combination of MET inhibitors (onartuzumab or crizotinib) and inhibitors to one of the upregulated tumorigenic proteins (mTOR, FGFR1, EGFR, STAT3, and COX-2) in these cells. When cells were treated with combination therapy, the rate of cell death increased, whereas the rate of cell proliferation decreased compared with single-agent treatment. In addition, mice bearing the MET-inhibitor-resistant GBM orthotopic xenografts treated with both COX-2 or FGFR and MET inhibitors exhibited enhanced MET sensitivity that correlated with significant inhibition in tumor growth (Cruickshanks et al., 2018).

Olmez et al. (2018) reported on the significant activation of both c-Met and the TrkA-B pathways after CDK4/6 inhibition in GBM cell lines and discovered an interesting trans-activation network between the c-Met and TrkA-B pathways. As such, the authors simultaneously targeted both c-Met/TrkA-B (using altiratinib) pathways along with CDK4/6 (using abemaciclib) and observed a synergistic anti-tumor response with enhanced apoptosis after combination therapy. Interestingly, sole inhibition of CDK4/6 stimulated an NF-κB-induced upregulation of HGF, brain-derived neurotrophic factor, and nerve growth factor, which led to c-Met and TrkA-B pathway activation (Olmez et al., 2018), implicating the biochemical mechanisms by which these aggressive cancers attempt to evade therapeutic interventions. While this study was an adult GBM model, it is important to determine if this intricate network of trans-activation of these pathways may be present in pediatric nervous system tumors, further exacerbating the angiogenic potential of these cancers.

A fully human monoclonal antibody (AMG 102; rilotumumab) that targets the HGF/SF, exhibited anti-tumor activity against a xenograft model of U-87 malignant glioma model (Jun et al., 2007). A phase II study of AMG 102 in patients with recurrent GBM investigated its efficacy and safety in patients heavily pre-treated with bevacizumab compared with bevacizumab-naïve patients (Wen et al., 2011). No significant difference was reported in overall survival and anti-tumor activity with AMG 102 treatment in patients of either group. One possible explanation may be that AMG 102 is not a complete antagonist of HGF phosphorylation; therefore, c-Met signal transduction cascade may not be completely abrogated by AMG 102 as previously reported (Greenall et al., 2016). This observation may imply that combinatorial therapy to target alternate pathways that undergo compensatory upregulation and activation with this treatment may be beneficial.

Truffaux et al. (2015) compared dasatinib, an oral inhibitor of multiple targets (PDGFRA, PDGFRB, c-Kit, and SRC), monotherapy vs. combination therapy with cabozantinib, a potent inhibitor of c-Met, VEGFR2, and the proto-oncogene RET in a pre-clinical model of DIPG. Cell growth and invasion was decreased in primary DIPG cell lines treated with dasatinib monotherapy, but therapeutic effect significantly increased when combined with cabozantinib (Truffaux et al., 2015). Due to the multiple targeting effect of cabozantinib, it is difficult to conclusively attribute the results to c-Met inhibition; however, given the interactions discussed above, disrupting this interaction may be advantageous. A phase I clinical trial in pediatric DIPG combined dasatinib and MET inhibition with crizotinib, but failed to show promise in reducing tumor burden or prolonging life in these children (Broniscer et al., 2018). This is unfortunate especially considering that the preclinical studies showed promise when Dasatinib was combined with a c-Met inhibitor, cabozantinib, with increased antitumor effects *in vitro* using combination therapy versus single therapy (Truffaux et al., 2015).

Following the identification of gene therapy based on the noncoding small RNAs, Pang et al. (2019) studied the ability to create a viral-like particle with similar RNA interfering properties. The synthesized structure, dP@mVLP/RNAi_c-MET_, had the ability to cross the blood-brain barrier with the help of ApoEP and CPP surface modifications to downregulate c-MET induced DNA repair and promote the efficacy of oral temozolomide (TMZ) in an intracranial mouse model of GBM using U87 cells. The synergistic effect between dP@mVLP/RNAi_c-MET_ and TMZ was identified at multiple doses with complete cancer eradication with 5 mg/kg of dP@mVLP/RNAi_c-MET_ at a normal TMZ patient dose. This combination provides the potential for glioblastoma patients to have an enhanced, genetically regulated, and cost-effective treatment compared with ultrasound use or nanotherapeutic agents, which are currently used alongside TMZ. Animal trials, including the analysis of the liver and kidneys, which showed no signs of antigenicity or inflammation, solidified the therapeutic benefits of dP@mVLP/RNAi_c-MET_ pretreatment followed by oral TMZ compared with another synthesized molecule mVLP/RNAic-_MET_ pretreatment with TMZ, which proved ineffective, confirming gene therapy and chemotherapy combination as a unique and considerable approach to brain tumor treatment, which should be further explored (Pang et al., 2019).

In high-risk NBs, the inhibitor of HGF/c-Met signaling (EMD1214063) induced apoptosis and reduced proliferation in a panel of NB cell lines *via* inhibition of c-Met and MEK, phosphorylation. Moreover, *in vivo* xenograft models of the inhibitor retarded tumor growth thereby implicating EMD1214063 as a promising therapeutic target for high-risk NB *via* its antagonistic effect on the HGF/c-Met activation (Scorsone et al., 2014). The identification of c-Met as a clinically significant oncogenic pathway, especially pertaining to resistance to chemotherapeutic drugs, has led to the investigation of several drugs aimed to target this specific downstream pathway. Zhang et al. (2019) discussed the screening of multiple benzisoselenazolones (BISAs) in the inhibition of the c-Met signaling pathway. After rigorous screening and analysis of multiple BISAs, virtually all BISAs exhibited c-Met inhibitory and anti-tumor activity compared with Ebselen, a commonly known selenoorganic compound with antitumor and neuroprotective properties. The select structures found to have the highest activity were tested on other cell lines, including an NB line, and were observed to have similar effects on these cell lines, though each specific structure attained its anti-tumor activity through a different mechanism. BISAs containing thiourea, tetramethyl thiourea, or nitrogen heterocyclic groups had considerable effect on the autophosphorylation of c-Met and certain downstream products. It was recommended that the isothiourea and heterocyclic groups be added to Ebselen to enhance its activity. Knowing that c-Met type II inhibitors have multiple targets, this class of drugs may have other inhibitory pathways, which have yet to be discovered (Zhang et al., 2019).

Most of the studies described herein use compounds that target many pathways, and thus, we cannot conclude that the therapeutic effect was achieved *via* specific MET inhibition. To address the therapeutic efficacy of highly selective MET inhibitors, a highly selective MET kinase inhibitor, SAR125844, was tested in a phase I trial in patients with advanced solid tumors that harbored MET amplification. The maximum tolerated dose (MTD) was determined to be 570 mg/m^2^ administered once a week and showed significant anti-tumor activity in MET-amplified non-small cell lung carcinoma patients (Angevin et al., 2017; Choueiri et al., 2017).

Another highly selective small molecule inhibitor of MET (ANG 337) was tested in a phase I trial of patients with advanced solid tumors. AMG 337 was well tolerated with an MTD of 300 mg administered once daily that showed promising response rate in patient with MET-amplified solid tumors (Hong et al., 2019). Moreover, a highly selective MET tyrosine kinase inhibitor, savolitinib, was evaluated for its safety, pharmacokinetics, and anti-tumor activity in a first-in-human phase I trial in patients with advanced solid tumors (Gan et al., 2019) and was tested in a biomarker-based phase II trial of patients with advanced papillary renal cell carcinoma (PRCC; Choueiri et al., 2017). Savolitinib showed good tolerability and anticancer activity in the MET-driven cancers, thereby warranting further investigation of this compound in other MET-driven cancers. A more recent study, published by Choueiri et al. (2020) in JAMA Oncology, investigated the efficacy of savolitinib compared with sunitinib in the SAVOIR phase 3, open-label, randomized clinical trial of patients with MET-driven PRCC. Authors report a greater efficacy of savolitinib compared with sunitinib monotherapy with reduced grade 3 or higher adverse effects and reduced the need for drug dose modifications thereby encouraging further investigations into the use of savolitinib in this study population (Choueiri et al., 2020). Since none of these trials were conducted on nervous system cancers, their promising antitumor responses along with their good tolerability in patients in various other cancers warrants their further investigation in pediatric nervous system tumors.

### Therapeutic Targeting of c-Met in Cancer Stem Cells

In GBM, when HGF binds to MET and induces receptor dimerization and auto-phosphorylation, it triggers a cascade within the nucleus that signals for tumor growth, invasion, and metastasis. Piao et al. (2016) showed that evasive revascularization and recruitment of TIE2-expressing macrophages (TEMs) was driven by anti-VEGF therapy in GBM. The authors used a xenograft mouse model to study *in vivo* and *in vitro* activity of altiratinib, which inhibits MET/TIE2/VEGFR2 in human GSCs. Using GSCs (GSC17 and GSC267), altiratinib was shown to effectively reduce HGF-stimulated MET phosphorylation, which significantly reduced cell viability of GSC lines *in vitro*. More importantly, tumor volume and invasive potential were significantly reduced when altiratinib was combined with bevacizumab in an *in vivo*, xenograft model. In addition, the protein expression of mesenchymal markers as well as microvessel density, and TIE2-expressing monocyte infiltration were all significantly reduced after combination therapy compared with single therapy with bevacizumab. Furthermore, to determine the *in vivo* effect of combined therapy using altiratinib and bevacizumab in the GSC17 xenograft model, the authors reported significant increase in animal survival compared with bevacizumab mono-therapy (Piao et al., 2016). Therefore, the anti-proliferative, anti-invasive, and anti-angiogenic effects of altiratinib and its suppression of myeloid cell infiltration in GBM make it a potential therapeutic drug used as a single agent therapeutic or as a combinatory therapy with bevacizumab and may address the therapeutic resistance observed with mono-therapy.

Therapeutic failure in high-risk nervous system tumors may be due to radiotherapy resistance, usually accompanied by tumor recurrence and rapid growth that leads to mortality in these patients. It has been well established that GSC radio-resistance relies on efficient activation of the DNA damage response (Pal et al., 2018); however, the exact mechanisms are still unclear. One study revealed that the MET receptor kinase was specifically expressed in a subset of radio-resistant GSCs and overexpressed in human GBM that recur post radiotherapy. The authors reported that MET enhances GSC radio-resistance *via* a novel mechanism involving AKT activity through activation of Aurora kinase A, ATM kinase, and the downstream effectors of DNA repair. In addition, there was enhanced phosphorylation and cytoplasmic retention of p21, an anti-apoptotic protein thereby enhancing GSC survival. Furthermore, mice xenografted with GSCs to develop GBM and subsequently treated with MET inhibitors exhibited an accumulation of DNA damage in irradiated GSCs and their subsequent depletion (De Bacco et al., 2016). While MET overexpression and activation by HGF does not warrant its designation as a pivotal CSC marker, these studies demonstrate a role played by MET signaling that enhances therapeutic resistance and stem cell maintenance in these cancers. This highlights the importance of utilizing MET inhibitors to radio-sensitize tumors by reducing the malignant nature of the radio-resistant GSCs thereby increasing the possibility of long-term, cancer-free cures.

Undoubtedly, multiple target inhibition is crucial in reducing therapeutic resistance in nervous system tumors. However, the molecular mechanisms of the network between signaling pathways, which affect the response of cancer cells to targeted therapies are not completely understood. The role played by the TGF-*β* family and HGF/c-Met signaling in GBM pathogenesis is well established; however, characterization of their functional interactions remains an explorative avenue. Papa et al. (2017) observed that TGF-β reduces c-Met phosphorylation in human GSCs using genetic and pharmacological approaches to either stimulate or inhibit the TGF-β pathway. This effect was diminished after inhibition of either MAPK/ERK or PI3K/PKB/AKT signaling pathway. Studies comparing the c-Met-driven and c-Met-independent GCS models showed that TGF-β inhibits the maintenance of GSCs partly through its antagonism of c-Met activity. Importantly, human GBM and *ex vivo* single-cell gene expression profile studies using immunohistochemical analyses revealed a negative interaction between TGF-β and HGF pathways (Papa et al., 2017). This implicates the importance of the delicate balance between these two oncogenic pathways that may promote CSC maintenance.

While the majority of these studies were conducted on adult GBM models, and pediatric brain cancers differ vastly from adult brain cancers, there are common targets between the two. For example, while pediatric brain cancers are largely driven by epigenetic dysregulation and micro-environmental influences exert a greater effect on adult brain cancers, common molecular drivers between the two include MYCN, NOTCH, PI3K/AKT/MAPK/mTOR, WNT/−catenin, and SHH. In addition, common epigenetic aberrations between adult and pediatric brain cancers include SOX2, BMI1, EZH2, FOXG1, and STAT3 (Abou-Antoun et al., 2017), which are typical signatures seen in brain CSC propagation, maintenance, self-renewal propensity, and therapeutic resistance. As illustrated in Figure 3 and outlined in Table 1, string functional protein networks revealed experimentally determined interactions between MET and several of these molecules, specifically, between MET and SOX2, STAT3, PIK3CA, MYC, MYCN (*via* SOX2), and AKT1 *via* TNF. This analysis revealed highly tumorigenic, pro-angiogenic, pro-invasive/pro-metastatic/pro-proliferative/anti-apoptotic, and stem cell pluripotency pathways. Therefore, while adult and pediatric brain cancers do differ, the common players that cross-talk with HGF/MET are potential avenues for further investigation in order to delineate the cooperative or synergistic interactions between the said molecules in pediatric brain cancers and develop new treatment approaches. Future attempts at therapeutic and genetic intervention should consider the simultaneous, multi-modality targeted approaches to reach better prognostic outcomes in children with these aggressive tumors. As illustrated in Table 2, c-Met has been involved in various tumorigenic and stem cell-enriching pathways that may be aggravating these malignant tumors and warrants further investigation into therapeutic targeting of these pathways.

**Table 2:** Summary of the tumorigenic roles played by c-Met.

Stemness pathways	c-Met is a direct transcriptional target of WNT/β-catenin. MET is co-expressed with CD133 and/or CD15. MET-overexpression correlates with higher clonogenic survival. c-Met expressing cells were preferentially localized in perivascular regions GSCs. Stimulation of c-Met signaling pathway increased expression of Oct4, Nanog, and Klf4. HGF induced c-Met activation was most pronounced in the SHH subgroup of medulloblastoma.
Epigenetic dysregulation	JARID1B activates c-Met in cancer stem cells. c-Met and its ligand HGF/SF are involved in epigenetic dysregulation. SPINT2/HAI-2, an inhibitor of HGF/c-Met signaling, was silenced by promoter methylation in medulloblastoma.
Aberrant signaling	VEGFR cross-talks with c-Met. c-MET cross-talks with IGF1R, TGF-β, and EGFR. c-Met activation is mediated *via* the PI3K-AKT and RAS-MAPK. c-Met activation induces CDC42. c-Met and TrkA-B pathways trans-activate each other. PDRX1 expression stabilizes c-Met. TGF-β inhibits stemness in GSCs partly through its antagonism of c-Met activity. TNF-α induces MET transcription to sustain MEK/ERK activation and promote invasive growth.
Therapeutic resistance	MET enhances GSC radio-resistance *via* AKT activity through activation of Aurora kinase A, ATM kinase, and the downstream effectors of DNA repair. Sole inhibition of CDK4/6 led to a NF-κB-mediated upregulation of hepatocyte growth factor, brain-derived neurotrophic factor, and nerve growth factor, which further activates both c-Met and TrkA-B pathways. Treatment with altiratinib (inhibitor of MET/TIE2/VEGFR2) in human glioma stem cells inhibited expression of mesenchymal markers, microvessel density, and TIE2-expressing monocyte infiltration. Foretinib overcomes entrectinib resistance associated with the *NTRK1*-G667C mutation in *NTRK1* fusion-positive tumors.

Summary of the tumorigenic roles played by c-MET signaling on cancer stemness maintenance, epigenetic dysregulation, aberrant signaling, and therapeutic evasion. Abbreviations, c-MET, mesenchymal epithelial transition factor; WNT/β-catenin, wingless-related integration site/β-catenin pathway; CD 133, CD15, transmembrane phosphoglycoprotein protein 133 and 15; GSC, glioma stem cells; Oct 4, octamer-binding transcription factor 4; Nanog, transcription factor that is involved in the self-renewal of embryonic stem cells; Klf 4, Krüppel-like factor; JARID 1B, Jumonji C-domain-containing histone demethylase 1B; HGF/SF, hepatocyte growth factor/scatter factor; SPINT2/HAI-2, serine peptidase inhibitor, Kunitz type 2/hepatocyte growth factor activator inhibitor-2; VEGFR, vascular endothelial growth factor receptor; IGF 1R, insulin-like growth factor 1 receptor; TGF-β, transforming growth factor β; EGFR, epidermal growth factor receptor; PI3K-AKT, phosphoinositide 3-kinase-AK strain transforming; RAS-MAPK, RAS-mitogen-activated protein kinase; CDC42, cell division control protein 42; TrKA-B, tropomyosin receptor kinase A-B; PDRX1, peroxiredoxin 1; TNF-α, tumor necrosis factor α; MEK/ERK, MAPK/ERK kinase/extracellular receptor kinase; ATM kinase, ataxia-telangiectasia-mutated (ATM) protein kinase; CDK 4/6, cyclin-dependent kinase 4/6; NF-κB, nuclear factor κB; TIE 2, receptor tyrosine kinase 2; NTRK-1, neurotrophic tyrosine receptor kinase 1.

## Mechanisms of Therapeutic Resistance

While a plethora of pre-clinical studies have shown the efficacy of combined therapy to target various signaling pathways in nervous system tumors, acquired resistance to tyrosine kinase inhibitors or radiotherapy remain a hurdle in therapeutic intervention. Often the targeting of one signaling cascade in cancers was shown to induce the upregulation and activation of yet another tumorigenic pathway in a compensatory fashion. Examples include upregulation and activation of MAPK after mTOR inhibition in melanoma (Carracedo et al., 2008; Gopal et al., 2014); activation of c-Met and upregulation of matrix metalloproteinase and hypoxia-inducible factor 1α in glioblastoma (Mahase et al., 2017); the upregulation of the L1 cell-adhesion molecule (L1-CAM) and MYCN after radiotherapy in neuroblastoma (Rached et al., 2016); the compensatory upregulation of heat shock proteins (HSPs), prohibitin, fatty acid-binding protein 5 (FABP5), and high mobility group A1 (HMGA1) in NB stem cells after radiotherapy (Zaatiti et al., 2018), and finally, the compensatory upregulation of p-L1CAM, MYCN, and prohibitin after HSP90 inhibition in MYCN-amplified human neuroblastoma cells (Hanna et al., 2021). Unfortunately, clinical trials have yet to report on an effective treatment that eradicates CSCs and bulk cancer cells in these tumors, leading to long-term, cancer-free survival (King et al., 2017), possibly due to the impermeable BBB and the efficient clearance and elimination of small molecules from the central nervous system. GBM and DIPG therapeutic resistance has been the major hurdle in treatment failure and mortality. Much concern is put on various resistance mechanisms such as upregulation of ABC drug-efflux proteins (Veringa et al., 2013) that effectively clear out small molecule therapeutics, overexpression of DNA repair molecules (Chornenkyy et al., 2015), evasion of apoptotic signals (Wu et al., 2017), and particularly, the evasive properties of the CSC population in these tumors (Kumar et al., 2017). This subset of aggressive cells is implicated in the therapy resistance and subsequent relapse with metastatic disease in these patients. The role of MET in this scenario has been well reported in GBMs and other tumors. In addition, the drug delivery method used to date in these tumors may not be ideal in inducing complete tumor infiltration. Administering therapeutic compounds systematically may be problematic considering these are brain tumors, which are protected by the selective permeability of the BBB (Himes et al., 2019) that may be a hindrance to their penetration into the bulk tumors. As such, convection-enhanced delivery methods that potentially bypass the BBB and nanoparticle-delivery of radio sensitizers (King et al., 2017) may address this concern and yield better tumor infusion of the therapeutic agent administered.

An alternative approach to c-Met-targeted treatment was the downregulation of the c-Met-induced DNA repair pathway to allow improved effect of chemotherapeutic agents. Wu et al. (2019) identified a mechanism of TMZ resistance through long non-coding RNA (lnc-RNA) as a crucial focal point for new therapeutic interventions. The lnc-RNA specific to GBM was established to be lnc-TALC whose increased expression was directly correlated with elevated resistance to TMZ and poorer prognosis. The phosphorylation of AKT was among the aberrant pathways of lnc-TALC; hence, its inhibition led to the autophagy and apoptosis of TMZ-resistant GBM. The expression of lnc-TALC was determined to be the promotion of AKT, which induced the degradation of the FOXO transcription factor resulting in drug resistance. Elevated levels of c-Met were associated with recurrence and poorer prognosis resulting from the mesenchymal transformation of endothelial cells, which made them more resistant to TMZ (Wu et al., 2019). A fourth pathway of lnc-TALC is its competitive binding to micro RNA sites, specifically its competitive binding with miR-20b-3p. Furthermore, the MGMT pathway was linked with TMZ resistance and poor prognosis owing to its potential loss of hypermethylation and increased rate of DNA repair, but the lnc-RNA did not show any effect on the methylation of MGMT promoter and did not affect that aspect of TMZ resistance or recurrence of GBM (Wu et al., 2019). Additionally, the regulation of STAT3, a downstream transducer of c-Met, showed decreased TMZ resistance, and though increased STAT3 prompts MGMT expression, the former’s inhibition was not associated with MGMT regulation implying that the regulation is independent of its transcriptional activity (Wu et al., 2019). Lnc-TALC are certainly potential targets in future therapies as a result of their AKT-mediated TMZ resistance in GBM (Wu et al., 2019).

A more recent investigation revealed that metabolic reprograming may lead to therapeutic resistance to c-Met-targeted therapy observed in clinical trials. Zhang et al. (2020) sought out to determine the mechanisms that drive treatment evasion to c-Met inhibitor using gene set enrichment and proteome analysis coupled with metabolite screening by chromatography/mass spectrometry. Interestingly, the authors reported significant metabolic reprogramming elicited by c-Met inhibition including activation of oxidative metabolism in established and patient-derived xenograft cells, increased reactive oxygen species production, enhanced oxygen consumption rate, and an increase in mitochondria. All these changes were affiliated with upregulation of the master-regulator transcription factor, PGC1α, leading to fatty acid oxidation and glucose anaplerosis (Zhang et al., 2020).

More importantly, the authors were able to suppress this response to c-Met inhibition (using crizotinib) by combining it with the potent mitochondrial matrix chaperone inhibitor, gamitrinib. The combination therapy reduced oxidative stress and led to potent anti-tumor activity evidenced by synergistic cell death compared with single-dose treatment with either compound alone (Zhang et al., 2020). This unique study combined high-throughput analysis of genomic, transcriptomic, and metabolomic data to derive the ideal drug-drug combination in order to successfully overcome resistance to single-target inhibition and reach better, long-term cures for patients with these aggressive cancers. Similar studies would be highly encouraged in pediatric nervous system tumor models in order to hone in on the most optimal therapeutic regime to deliver highly targeted, non-toxic therapeutics to children with these devastating diseases.

Applying c-Met inhibition on pediatric cancers has been slightly more challenging. Broniscer et al. (2018) conducted a clinical study for the combined use of dasatinib and crizotinib for the treatment of progressive HGG and DIPG. While the outlook toward finding a potent treatment for these gliomas was encouraging given the tumor’s refractory nature, the combination of dasatinib and crizotinib was poorly tolerated by the patients with some severe unexpected adverse effects including proteinurea in the nephrotic stage and hyponatremia, though no radiological effects were noted (Broniscer et al., 2018). Although dasatinib did cause a decline in p-AKT levels, the concentration of the drug crossing the BBB was insufficient to cause a significant response, and its binding to receptors besides PDGFRA may be responsible for its multiple toxicities. Crizotinib was also found to have inefficient BBB penetration resulting in low c-Met inhibition despite it being the only commercially available c-Met inhibitor at the time of the study. In brief, while there is no further need to study the potential benefits of crizotinib and dasatinib combination, more work on pediatric brain tumors should be conducted to identify proper dosing and benefits of each drug separately. More importantly, mechanisms that increase the BBB penetrance of these compounds may prove beneficial in inducing anti-tumor activity in this group of patients.

## Conclusion

Malignant nervous system tumors in children are devastating diseases with current therapies being largely ineffective, possibly due to the highly invasive, treatment-resistant, and recurrence-prone stem cell sub-populations within the bulk tumors. Epigenetic dysregulation, aberrant signaling, therapeutic resistance, and the intricate interplay between c-Met and other tumorigenic drivers may fuel the aggressive nature of these cancers (Table 1). The ability of the aggressive cancer cells to undergo EMT, possibly driven by c-Met and its cross-talk with other EMT-driving molecules, and manipulate their micro-environmental niche to their advantage are key factors that must be effectively targeted. Therapeutic resistance and recurrence may be eradicated if therapeutic interventions were aimed at multiple pathways that play pivotal roles in this phenotype. The observed interaction of c-Met with many of the tumorigenic pathways discussed in this review that drive treatment resistance, EMT and CSC maintenance, angiogenesis, proliferation, and invasion/metastasis in various tumors implicates c-Met as being one of the key players in cancer malignancy and that multi-modality targeted therapy aimed at inhibiting these redundant and compensatory pathways may show promise in therapeutic efficacy.

## Author Contributions

TA-A conceptualized the study, designed the figures, and wrote the original draft of the study. TA-A and AK reviewed and initially screened the literature and performed the revisions and editing on the final manuscript. Both the authors contributed to the article and approved the submitted version.

### Conflict of Interest

The authors declare that the research was conducted in the absence of any commercial or financial relationships that could be construed as a potential conflict of interest.
